# Comparison study of gastrinomas between gastric and non-gastric origins

**DOI:** 10.1186/s12957-015-0614-6

**Published:** 2015-06-16

**Authors:** Song-Fong Huang, I-Ming Kuo, Chao-Wei Lee, Kuang-Tse Pan, Tse-Ching Chen, Chun-Jung Lin, Tsann-Long Hwang, Ming-Chin Yu

**Affiliations:** Department of Surgery, Chang Gung Memorial Hospital, 5, Fu-Shin Street, Kweishan, Taoyuan 333 Taiwan; Department of Medical Imaging and Intervention, Chang Gung Memorial Hospital, 5, Fu-Shin Street, Kweishan, Taoyuan 333 Taiwan; Department of Pathology, Chang Gung Memorial Hospital, 5, Fu-Shin Street, Kweishan, Taoyuan 333 Taiwan; Department of General Gastroenterology, Chang Gung Memorial Hospital, 5, Fu-Shin Street, Kweishan, Taoyuan 333 Taiwan

**Keywords:** Gastrinoma, Lymph node metastasis, Liver metastasis, Prognosis

## Abstract

**Background:**

Gastrinomas are one of the neuroendocrine tumors with potential distant metastasis. Most gastrinomas are originated from pancreas and duodenum, but those of gastric origin have been much less reported. The aim of the study is to compare gastrinomas of gastric and non-gastric origins.

**Methods:**

Four hundred twenty-four patients with neuroendocrine tumor by histological proof in Chang Gung Memorial Hospital, Linkou branch in the past 10 years were included. A total of 109 (25.7 %) cases were identified of upper gastrointestinal origins, of which 20 (18.3 %) were proven gastrinomas. The clinical characteristics were collected and analyzed retrospectively.

**Results:**

In our study, 21 tumors of the 20 cases were identified by pathologic proof, 11 (55 %) had resection or endoscopic mucosa resection, 9 of gastric origins, 9 of duodenal origins, 2 of pancreatic origins, and 1 of hepatic origins. One case had multiple lesions. Patients with gastric gastrinomas had older age, higher levels of gastrin, seemingly smaller tumor size (*p* = 0.024, 0.030, and 0.065, respectively), and usually lower grade in differentiation (*p* = 0.035). Though gastric gastrinomas had a high recurrent rate (80 %), the lymph node and liver involvement was less common. Gastrinomas with liver involvement/metastasis had a high mortality rate where 80 % died of liver dysfunction.

**Conclusions:**

Gastrinomas originating from stomach had higher gastrin level and lower tumor grading and presented at older age. The long-term outcome was probably better than non-gastric origin because of lower grading and less lymph node and liver involvement.

## Background

Gastroenteropancreatic neuroendocrine tumors are differentiated from epithelial cells of gastrointestinal tract or pancreas. Even though less common than other gastrointestinal malignancy, the incidence of neuroendocrine tumors has significantly increased in the last three decades because of the advance in diagnosis [1–8]. The gastroenteropancreatic neuroendocrine tumors can secrete different kinds of neuropeptides or hormones, leading to a wide spectrum of clinical presentations, ranging from completely asymptomatic to patient discomfort. The neuroendocrine tumors can be grouped into functional or nonfunctional by clinical presentations [2]. Gastrinomas, one of the common functional neuroendocrine tumors, can over-synthesize gastrin and are usually presented as recurrent peptic ulcer disease, gastroesophageal reflux disease, diarrhea, or abdominal pain and are referred to as Zollinger-Ellison syndrome (ZES) [9–13].

More than 80 % of gastrinomas are found in the gastrinoma triangle, an important anatomic mark for localization [14–16]. Advances in imaging tools, such as computed tomography and magnetic resonance imaging with dynamic phases, help identify the location of gastrinomas. Recently, endoscopic ultrasound and somatostatin receptor scintigraphy have provided better sensitivity for subcentimeter gastrinomas [10]. But the incidence of gastric gastrinomas was increased in the past 50 years [2]. Gastrinomas in the pancreas have higher malignancy rate as compared to those in the duodenum [17]. The therapeutic strategies for gastrinomas include surgical treatment, local endoscopic mucosal resection (EMR), targeted therapy, chemotherapy, radiotherapy, and acid antisecretory medication [18]. Surgery and local resection, with an intent for curative treatment, still has more than 30 % of recurrence rate, where lymph nodes and livers are the most common metastatic sites for relapse (in 86 and 43 % of cases, respectively) [7, 14, 19–21]. Diffused liver tumor infiltration carries the worst survival rate, and multiple lymph node metastases are associated with rapid tumor relapse [18]. However, in one report, curative resection and regional lymph node dissection provided better prognosis [19].

In recent years, increasing incidence of subclinical gastric gastrinomas has been found by panendoscopic examination. Gastric NETs are divided into four groups [22]. Type 1 and type 2 are gastric gastrinomas, which cause hypergastrinemia and can be treated by local excision, such as endoscopic submucosal dissection or endoscopic polypectomy, but partial or total gastrectomy may be needed when recurrence occurs [23, 24]. Type 3 and 4 are rare and not associated with gastrin production. However, the difference of clinical pictures and outcomes among gastric, pancreaticoduodenal, and hepatic gastrinomas has not been well reviewed from the literature, except for a few studies of gastrinomas in eastern Asia [7, 23]. In this study, we performed a clinical cohort review of gastrinoma cases in a single institute, especially for these two different origins.

## Methods

### Patients

A retrospective cohort study from 1 January 2002 to 31 December 2012 was approved by the Institutional Review Boards, Chang Gung Memorial Hospital (IRB 102-2975B). Four hundred twenty-four patients with pathologically proven neuroendocrine tumors from the Cancer Registry of Cancer Center, Chang Gung Memorial Hospital, Linkou branch were checked of histology, and 109 (25.7 %) cases with tumors of upper gastrointestinal origins were selected. The diagnosis of gastrinomas was based on clinical symptoms as Zollinger-Ellison syndrome, higher serum gastrin level, and pathology criteria [25, 26]. Of these, 20 patients (18.3 %) were enrolled, and the demographic data, peptic ulcer/GERD treatment, gastrin level, tumor size, tumor grading, and metastasis were recorded, in which 9 specimens were of gastric origin, 9 specimens of duodenal origin, 2 of pancreatic origins, and 1 of hepatic origin. One patient had multiple lesions. Most were sporadic gastrinomas, but two cases from the same family were MEN type 1. The treatment methods including resection, endoscopic mucosal resection, and adjuvant/salvage treatment were analyzed.

All patients had imaging studies, at diagnosis and follow-up, using endoscopic ultrasound (EUS), computed tomography (CT), or magnetic resonance imaging (MRI). Patients were divided into two groups according to their primary site of origin (gastric or non-gastric), and continuous and categorical data were analyzed by Student’s T test and chi-square methods. The overall survival was analyzed by log-rank test, and significant difference was defined as *P* value < 0.05. (IBM SPSS Statistics 20.0.)

### Immunohistochemistry

Formalin-fixed and paraffin-embedded resection specimens were sectioned to 4 μm in thickness and deparaffinized, rehydrated, and processed for antigen retrieval. The slides were further incubated with appropriate dilutions of the following antibodies at room temperature for 1 h. After incubation, the slides were washed three times in phosphate-buffered saline (PBS), incubated with a horse reddish peroxidase conjugated antibody polymer (Zymed) at room temperature for 10 min, and were then developed by treatment with 3,3′-diaminobenzidine (Roche) at room temperature for 10 min. The used monoclonal antibody for Immunohistochemistry (IHC) were against Ki-67 (1:1000, Dako) and chromogranin A (1:500, Dako). For gastrin staining we used polyclonal antibody against gastrin (LEICA biosystems) without antigen retrieval. Ki-67 staining and mitotic counts per 10 high power field were used for tumor grading [25]. In brief, more than 20 in mitotic index were classified as G3. Independent experienced pathologists blinded of patient characteristics and outcomes studied the results of immunohistochemical staining for diagnosis and classification [25].

## Results

The details of 20 cases are shown in Table 1. The mean age was 47.2 years with equal gender distribution. The mean tumor size was 7.3 mm, and the mean gastrin level at diagnosis was 352.0 pg/ml. Seven cases (36.8 %) developed recurrence and most had metastasis to liver and lymph node. Four cases were lost for follow-up because of liver metastasis and dysfunction. The mean follow-up period was 73.2 months. The demographic data are shown in Table 2.

**Table 1:** Personal characteristic data of twenty cases with gastrinoma

Case no.	Age	Gender	Clinical presentation	Primary site	WHO grading	Gastrin (pg/ml)	Procedure	Tumor size (mm)	Outcome	F/u period (months)	LN meta	Liver meta	Other meta	Medical treatment
01	51	Male	GERD, Erythematous gastritis	Stomach	G1	641	PES biopsy	6.0	Alive with regional tumor	65.6	Nil	Nil	Nil	H2 blocker
02	52	Male	Epigastralgia, Erythematous gastritis, Diarrhea	Stomach	G1	924	PES biopsy	2.0	Alive with regional tumor	111.5	Nil	Nil	Nil	Nil
03	50	Male	Epigastralgia	Stomach	G1	568	EMR	4.0	Free	127.8	Nil	Nil	Nil	Nil
04	75	Female	Epigastralgia, Erythematous gastritis	Stomach	G1	993	PES biopsy	N/A	Alive with regional tumor	27.6	N/D	N/D	N/D	Nil
05	74	Female	GERD, Gastric erosions	Stomach	G1	2878	PES biopsy	6.0	Alive with regional tumor	32.6	N/D	N/D	N/D	Nil
06	48	Female	Z-E syndrome, Diarrhea	Stomach	G1	671	Total gastrectomy	N/A	Liver recurrent	77.2	Nil	7 mm, S5/8	Nil	Nil
07	58	Female	UGI bleeding	Stomach	G1	1690	EMR	4.8	Recurrent, site unknown	80.4	Nil	Nil	Nil	H2 blocker
08	56	Female	GERD, Erythematous gastritis	Stomach	G1	1770	EMR	8.0	Regional recurrent	94.8	Nil	Nil	Nil	H2 blocker
09	43	Female	Duodenal ulcer, GERD grade A	Stomach	G1	1817	EMR	5.2	Regional recurrent	112.3	Nil	Nil	Nil	H2 blocker
10	12	Male	Diarrhea, Esophageal ulcers, Gastric ulcer	Duodenum	G3	8219	Liver biopsy PES biopsy	N/A	Expired—liver failure	9.44	Yes^a^	Yes	Bone Lung	Octreotide LAR PPI Evolimus CT^c^
11	35	Male	Repeat peptic ulcer, Epigastralgia	Duodenum	G1	1179	Antrectomy + Duodenectomy with lymph node resection	15.0	Alive with lymph node metastasis	26.2	Yes, Group 12	Nil	Nil	Octreotide LAR PPI
12	64	Male	Chronic gastritis	Duodenum	G1	75	EMR	5.0	Free	42.9	N/D	N/D	N/D	Nil
13	32	Male	GERD grade A Superficial gastritis, Duodenal erosions	Duodenum	G1	58	PES biopsy^e^	5.0	Alive with disease (MEN-1)	48.2	Nil	Nil	Nil	Nil
14	34	Male	Epigastralgia	Duodenum and pancreas	G1	35	Subtotal gastrectomy + distal pancreatectomy^f^	6.0	Alive with disease (MEN-1)	53	Yes^b^	Nil	Nil	Octreotide LAR Evolimus PPI
15	39	Male	Gastric ulcers	Duodenum	NA	105	PES biopsy	8.0	Alive with regional tumor	127.3	N/D	Nil	N/D	PPI
16	25	Male	Diarrhea	Pancreas	G2	359	Liver biopsy	N/A	Expired	64	Yes	Yes	Nil	Octreotide LAR PPI Sunitinib
17	55	Female	UGI bleeding, Peptic ulcer	Duodenum	G2	nil	Liver biopsy	N/A	Expired	27.1	Yes	Yes	Yes	PPI
18	72	Female	Diarrhea, Abdominal pain	Duodenum	NA	1341	EMR	15.0	Free	80.3	Nil	Nil	Nil	PPI
19	36	Female	GERD	Duodenum and lymph node	G1	217	LN excision EMR	60.0	Free	102.1	Yes	Nil	Nil	Nil
20	33	Female	Peptic ulcer, UGI bleeding	Liver	G3	1570	Hepatectomy	150.0	Expired—liver failure	205.8	Nil	Liver origin	Bone PC	Octreotide LAR HAIC^d^ PPI

*N/A* not available, *ND* not done, *PES* panendoscopy, *EMR* endoscopic mucosal resection, *LAR* long-acting repeatable, *PPI* proton pump inhibitor, *CT* chemotherapy, *HAIC* hepatic arterial infusion chemotherapy, *GERD* Gastroesophageal reflux disease, *PC* Peritoneal carcinomatosis

^a^Mediastinal and Porta hepatis lymph nodes

^b^Mediastinal lymph nodes

^c^Chemotherapy with VP-16/Etoposide + Cisplatin/CDDP

^d^HAIC with 5-FU + Cisplatin

^e^Specimen positive of gastrin and somatostatin

^f^Positive of gastrin, glucagon, and insulin

**Table 2:** Gastric origin compared with non-gastrin origin

	All	Gastric	Non-gastric	*P* value
	*n* = 20	*n* = 9 (45 %)	*n* = 11 (55 %)	
Age (years)	47.2 ± 16.8	56.3 ± 11.2	39.7 ± 17.4	0.024
Gender (M/F)	10/10	3/6	7/4	0.370
Size (mm)	7.3 ± 4.3	5.1 ± 1.9	9.1 ± 5.0	0.065
Grading by WHO G1/G2/G3^a^	14/2/2 (78 %/11 %/11 %)	9/0/0	5/2/2	0.035
Gastrin level (pg/ml)	352.0 ± 313.7	512.3 ± 323.3	207.7 ± 234.7	0.030
Treatment (Biopsy/resection)	9/11 (45 %/55 %)	4/5 (44 %/56 %)	5/6 (45 %/55 %)	1.000
Metastasis^b^	7 (36.8 %)	1 (12.5 %)	5 (45.5 %)	0.080
Recurrence^c^	7 (63.6 %)	4 (80 %)	3 (50 %)	0.348
Death, disease specific	4 (20 %)	0 (0 %)	4 (36.4 %)	0.068

^a^Two cases of biopsy were not suitable for histology grading

^b^One case has no enough follow-up

^c^9 cases had biopsy and no treatment

**Fig. 1:**
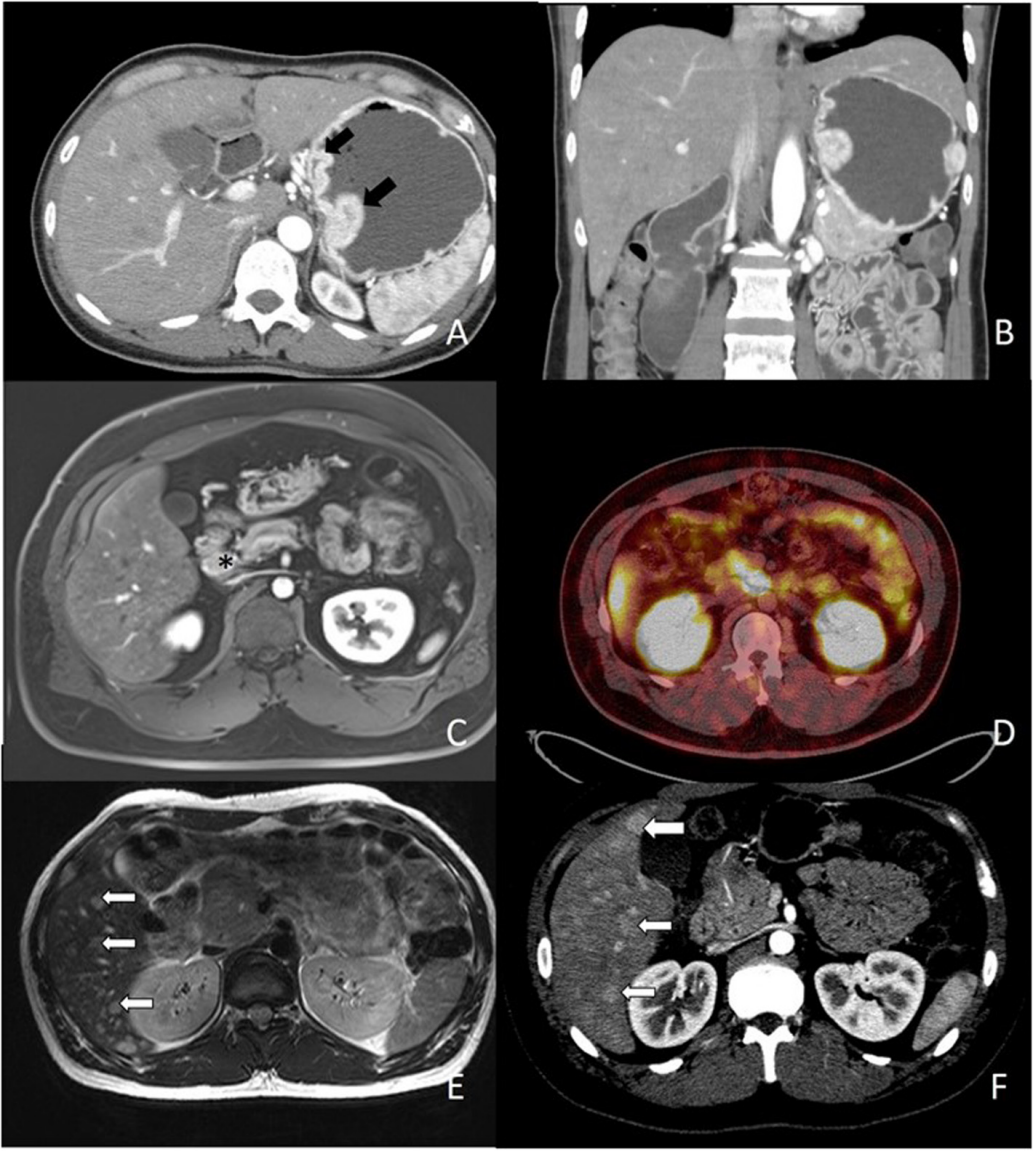
Axial contrast enhanced CT scan of upper abdomen (arterial phase) shows **a** two enhanced polypoid mass (*black arrow*) at high gastric body along lesser curvature side. **b** Coronal multiplanar reformation image. (1) Axial dynamic fat-saturated T1-weighted image with gadolinium enhancement shows enlarged lymph node (*) at peripancreatic area (**c**). (2) Ga-68 DOTATOC-scan shows strong uptake at medial aspect of uncinate process of pancreas (**d**). (3) Infiltrative tumor at pancreatic head with diffuse liver metastases (*white arrow*) **e** Axial dynamic fat-saturated T2-weighted image with gadolinium enhancement. **f** Axial contrast CT scan in arterial phase

**Fig. 2:**
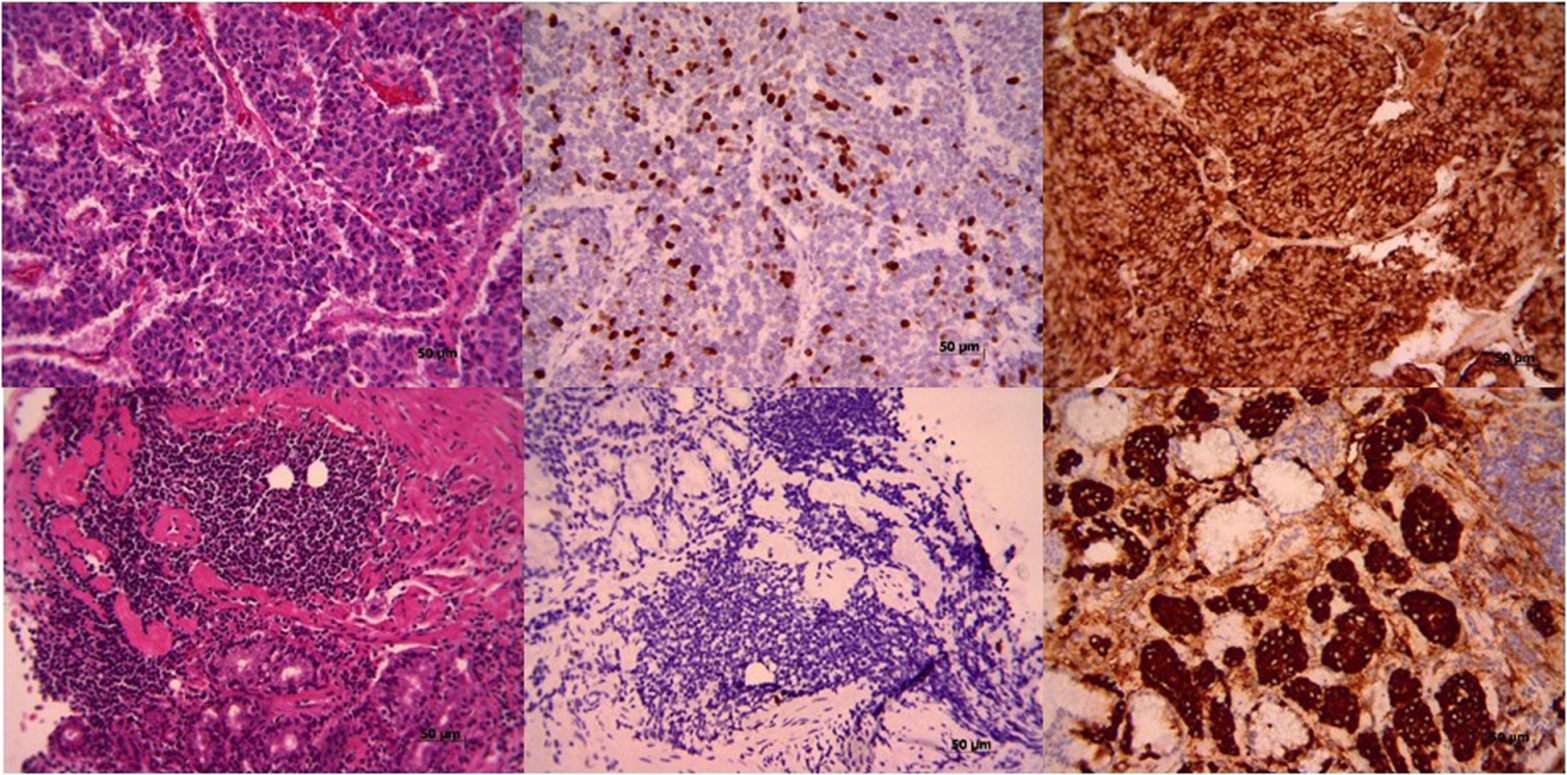
Histology and immunohistochemistry staining of gastrinomas. Upper panel represents high-grade non-gastric and lower panel is low-grade gastric gastrinoma. The image shows histology, Ki-67 staining, and chromogranin staining from left to right, (100× magnification)

**Fig. 3:**
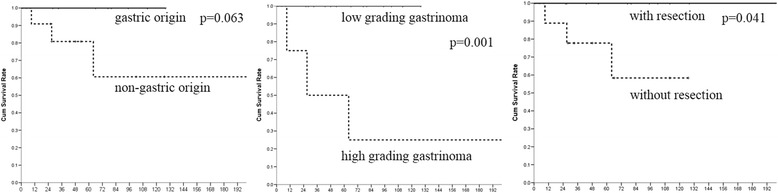
The Kaplan-Meier overall survival rate between **a** gastric origin (*solid line*) and non-gastric origin (*dotted line*), **b** low and high grading gastrinoma (G1 vs. G2, G3 solid line vs. dotted line), and **c** with/without resection (*solid line*/*dotted line*). *P* = 0.063, 0.001, and 0.041, respectively. WHO grading was most important for prognosis, but gastric gastrinomas got better survival rate

The CT imaging was shown in Fig. 1, with an emphasis on the presentation of lymph node metastasis. Compared with those of non-gastric origin, patients with gastric gastrinomas had significantly elder age (56.3 ± 11.2 vs. 39.7 ± 17.4, *p* = 0.024) and higher gastrin level (512.3 ± 323.3 vs. 207.7 ± 234.7 mg/ml, *p* = 0.030), however, smaller tumor size (7.3 mm vs. 5.1 mm, *P* = 0.065). Four gastrinomas of high or moderate tumor-grade (G2, 3) all had non-gastric origins (*p* = 0.035). The histology, Ki-67 staining, and chromogranin A staining are shown in Fig. 2. Overall survival rate was showed in Fig. 3. Patients with low grade gastrinoma and with resection treatment had better survival outcome. Besides, of those patients with tumor of gastric origin, although most received local treatment, seems to have less distant metastasis and better long-term survival; no statistically significant difference was found due to small case numbers.

## Discussion

Most gastrinomas are well-differentiated endocrine tumors with benign or low-grade malignant behavior, whereas poorly differentiated neuroendocrine tumors (NETs) are rare (1–3 %) [25]. Early reports that have shown pancreatic NETs are usually larger than 1 cm and have higher risks for liver metastases [14, 27]. However, gastrinomas originating from stomach have seldom been studied [17, 23, 24]. In this study, 20 cases were identified with pathology proof, in which 9 were of gastric and 9 were of duodenal origins. One case with more than 10 years of follow-up was identified to have lesions only in the liver, and primary liver gastrinoma was classified. Comparing with those of hepatic-pancreatic-biliary origins, gastric gastrinomas had significantly higher gastrin levels but smaller tumor size, lower ki-67 proliferation index, and more well-differentiated tumors (G1). The long-term outcome seems better in gastric origin group that no cases had lymph node involvement and only one had liver metastasis.

All nine cases of gastric origin showed higher gastrin level, whereas three cases of non-gastric origin had normal gastrin level (<100 ng/ml) from our study. Though gastric gastrinomas type I and II had better long-term outcome, the recurrence rate was higher up to 80 % in this series. Regional recurrence was common and proton pump inhibitors were not administered because they all had good quality of life. Regional recurrence was possibly related to multicentricity or inadequate resection [28]. Patients would not like to have another following treatment, as the report from Li, et al [23]. However, gastric gastrinomas could be treated with repeated resection in selected conditions [24].

Until now, unknown primary origins and distal metastasis are of the greatest concern. The incidence of subcentimeter gastric origins was higher than that of reported by others, suggesting that upper gastrointestinal studies should be considered when unknown origins were encountered [29]. Delayed identification of tumor origin occurred in one of our patients. She underwent retroperitoneal lymph node resection initially, and the primary duodenal origin was found when routine endoscopy was done during follow-up. Identifying the primary origin is still of great challenge. The outcome analysis studies revealed that patients with lymph node involvement distant metastases had poorer outcomes. Though the use of long-acting octreotide has been the clinical practice, the satisfying outcome is still not supported by stronger evidence [10, 30]. One case of liver origin reported years ago received repeated resection for her liver tumors. She did not survive with LAR treatments because of liver dysfunction. In our series, liver metastasis presented the worst outcome and four out of five cases had mortality at follow-up. As reported in some studies, aggressive LN dissection/resection strategy improved patient’s outcome, and it was also compatible with our findings [31].

One special case in this study is a MEN-1 patient with duodenal and pancreatic gastrinoma. The patient’s histology and special staining were reviewed, and the duodenal lesion and one of four pancreatic NETs were positive for gastrin stain. But MEN-1 patients who had pancreatic gastrinoma were extremely rare, and metastasis should be considered [32]. Because we have no further study for tumor genetic heterogeneity, this case was assumed as duodenal and pancreatic gastrinoma.

## Conclusions

In summary, patients with gastric gastrinomas usually have older age, higher gastrin level, more subcentimeter tumor size, and significantly lower tumor grading. Endoscopic mucosal resection usually helps minimize the need for medication need, but local recurrence can be common. The probability of distant metastasis is low, and long-term outcome is better than those of duodenum, pancreas, and liver origins.
